# Modeling Hydrodynamic Charge Transport in Graphene

**DOI:** 10.3390/ma15124141

**Published:** 2022-06-10

**Authors:** Arif Can Gungor, Stefan M. Koepfli, Michael Baumann, Hande Ibili, Jasmin Smajic, Juerg Leuthold

**Affiliations:** Institute of Electromagnetic Fields (IEF), 8092 Zurich, Switzerland; stefan.koepfli@ief.ee.ethz.ch (S.M.K.); michael.baumann@ief.ee.ethz.ch (M.B.); hande.ibili@ief.ee.ethz.ch (H.I.); jasmin.smajic@ief.ee.ethz.ch (J.S.); leuthold@ethz.ch (J.L.)

**Keywords:** graphene, discontinuous Galerkin, finite element method, nonlinear modeling, hydrodynamic model, Tesla valve, computational semiconductors

## Abstract

Graphene has exceptional electronic properties, such as zero band gap, massless carriers, and high mobility. These exotic carrier properties enable the design and development of unique graphene devices. However, traditional semiconductor solvers based on drift-diffusion equations are not capable of modeling and simulating the charge distribution and transport in graphene, accurately, to its full extent. The effects of charge inertia, viscosity, collective charge movement, contact doping, etc., cannot be accounted for by the conventional Poisson-drift-diffusion models, due to the underlying assumptions and simplifications. Therefore, this article proposes two mathematical models to analyze and simulate graphene-based devices. The first model is based on a modified nonlinear Poisson’s equation, which solves for the Fermi level and charge distribution electrostatically on graphene, by considering gating and contact doping. The second proposed solver focuses on the transport of the carriers by solving a hydrodynamic model. Furthermore, this model is applied to a Tesla-valve structure, where the viscosity and collective motion of the carriers play an important role, giving rise to rectification. These two models allow us to model unique electronic properties of graphene that could be paramount for the design of future graphene devices.

## 1. Introduction

Graphene has been the focus of various research topics for the last two decades and is pushing toward the market [1,2], and even new physical effects such as Moiré materials with superconductivity [3,4] and chiral plasmons [5] are still being discovered. For the future of graphene and for the development of graphene-based devices, it is crucial to be able to understand its many effects and to model the electronic transport in graphene. Thanks to its 2-D structure, graphene has exceptional electronic transport characteristics such as zero-mass carriers at the Dirac point and very high charge mobility. These properties make it challenging to use traditional semiconductor-modeling techniques with graphene. Additionally, due to the collective behavior of charge carriers and the dominant electron–electron (e–e) collisions occurring in graphene, considerably high viscosity levels in carriers of graphene have been observed. The viscosity of electrons in graphene has been shown to lead to phenomena such as whirlpools and negative local resistance [6], superballistic transport [7], and Poiseuille flow [8,9]. When wisely utilized, these hydrodynamic effects could also be utilized to implement electronic devices that show rectifying properties. A 2-D Tesla valve has been implemented [10] that acts like a viscometer and demonstrates rectification. Graphene is considered as a semi-metal or a zero-gap semiconductor; however, its special attributes and zero band gap make it unique. Thus, the use of traditional semiconductor models, based on classical drift-diffusion equations, is challenging. There have been multiple attempts to extend the drift-diffusion solvers to model graphene-based devices [11,12,13,14,15], and they are valid for a range of applications; however, they lack the ability to fully capture the aforementioned phenomena, due to viscosity and the many-body interactions occurring with the carriers in graphene. Moreover, the existing hydrodynamic models for charge transport in graphene are limited to the relativistic case at 0 K [16]. Therefore, a generalized hydrodynamic study is required to model the motion of electrons in graphene and graphene-based electronic devices accurately. By considering mass, momentum, and energy conservation of the charge carriers in graphene, the charge-transport dynamics can be predicted [17,18,19]. In this work, we aim to demonstrate novel computational solvers and algorithms to model graphene’s electronic-transport properties, and we report two separate solvers based on the Finite Element Method (FEM). The first solver is to model the gating and contact doping in graphene, which solves the arising nonlinear Poisson’s equation iteratively to determine the Fermi level and charge densities in graphene layer. The second reported solver models the hydrodynamic charge transport in the time domain, based on the Discontinuous Galerkin Time Domain (DGTD-FEM) algorithm, and delivers dynamic charge transport in graphene in the time domain.

This article is structured as follows. Section 2 focuses on the theory and physical model to cover the nonlinear electrostatic equation to model the electrostatic gating and contact doping in graphene as well as the hydrodynamic transport. Section 3 shows the results arising from the nonlinear electrostatic solver, as well as the transient hydrodynamic transport solver that demonstrates Poiseuille flow and rectification in a graphene-based Tesla valve. Section 4 discusses the results, possible future work, and concludes the paper. 

## 2. Theory and Methods

### 2.1. Nonlinear Electrostatic Approach: Modeling Gating and Contact Doping

**Figure 1:**
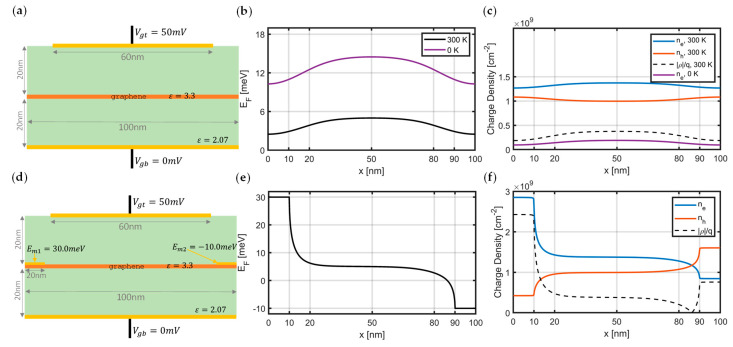
Electrostatic analysis of graphene layer with gating and contact doping. (**a**) Device geometry (cross-section) with top and bottom gates. Graphene is depicted with the orange layer in the middle, and the green layers represent dielectrics. (**b**) Fermi level profile on the graphene layer, when the top gate is kept at 50 mV and the bottom gate is grounded. Elevated Fermi level profile is obtained from the nonlinear Poisson’s equation using Thomas–Fermi distribution (1), 0 K case is obtained by assuming only electrons exist and $n_{e}^{2D}\left(E_{F}\right)=\frac{E_{F}^{2}}{\pi {\left(\hbar v_{F}\right)}^{2}}$, as in [21,22] (discussed further in the supplementary notes and Figure S1: 2-D device geometry (cross-section) with two gates at the top and the bottom. Graphene is depicted as orange layer in the middle, the green layers represent dielectrics, the gate contacts are shown in golden, and Dirichlet conditions are applied to those boundaries for solving the nonlinear Poisson’s equation.). (**c**) Corresponding 2-D charge (electron, hole, and total) distributions. (**d**) Scheme with two additional contacts on graphene, which pin the Fermi level at 30meV and −10meV, respectively. (**e**,**f**) Fermi level and charge distributions on graphene layer with the contact doping and gating present together. These profiles were directly obtained by the iterative solution of a nonlinear Poisson’s equation, together with Thomas–Fermi equation using the successive under-relaxation method.

The transport properties of graphene depend on many internal and external parameters, such as its deposition technique, impurities, type of materials in contact with graphene, operating temperature, etc. These aspects need to be accurately considered for the design of next-generation graphene-based electronic devices in order to account for the effects, such as nonlinear contact doping, due to metals. To exploit the full potential of graphene’s exotic properties, one should be able to access and manipulate the Fermi level of graphene. Making use of graphene in electrical devices requires shifting the Fermi level, by gating and making contacts with metals, which inherently dopes the graphene locally and results in Fermi level shifts. Both of these effects, gating and contact doping, can already be analyzed under static conditions where there is no net current flowing through the graphene. The key relations that determine the electrostatic charge and Fermi level distribution are the Thomas–Fermi equation and Poisson’s equation. These equations can be written for the analysis of charge distribution of graphene under electrostatic conditions. Using the equilibrium Fermi–Dirac function $f_{FD}\left(E-E_{F}\right)$, and 2-D density of states in graphene $g_{2D}\left(E\right)=\frac{2\left|E\right|}{\pi \hbar^{2}v_{F}^{2}}$, the electron density per area $n_{e}^{2D}$ at a given Fermi Level $E_{F}$ for nonzero temperature T, is written as

(1) $$n_{e}^{2D}\left(E_{F}\right)=\int_{0}^{\infty }g_{2D}\left(E\right)f_{FD}\left(E-E_{F}\right)dE=-\frac{2}{\pi }{\left(\frac{k_{B}T}{\hbar v_{F}}\right)}^{2}Li_{2}\left(-e^{\frac{E_{F}}{k_{B}T}}\right),$$

where $k_{B}$ is the Boltzmann constant and $Li_{n}\left(x\right)$ is the poly-logarithm function of the n-th order [12]. Electron-hole symmetry in graphene’s band diagram yields a similar relationship for the hole density $n_{h}^{2D}\left(E_{F}\right)=n_{e}^{2D}\left(-E_{F}\right)$. For devices where the graphene layer is sandwiched between dielectric layers, as in Figure 1, the following nonlinear Poisson’s equation can be written [11,14,15]:

(2) $$\nabla \left(\varepsilon \nabla \varphi \right)=-\rho \left(\varphi \right),$$

(3) $$\;\rho \left(\varphi \right)=\left\{\begin{array}{cc} q\left[n_{h}^{2D}\left(\varphi \right)-n_{e}^{2D}\left(\varphi \right)\right]/t_{gr} & ,\;\mathrm{within}\;\mathrm{graphene}\\ 0 & ,\;\mathrm{otherwise} \end{array}\right..$$

where φ is the electrostatic potential, ε is permittivity, q denotes elementary charge, and $t_{gr}$ is the graphene’s thickness, taken as 0.35 nm. Dividing by graphene’s thickness on the right-hand side of (3) makes sure that the volumetric charge density is used for the charge density term in Poisson’s equation. Here, Equation (2) represents the nonlinear Poisson’s equation (NLP), as the charge distribution in graphene is also a function of the potential. The obtained potential on the graphene layer shifts the band diagram of graphene based on E=−qφ, and the corresponding Fermi level with respect to the Dirac point can be updated. Therefore, solving this equation would require an iterative approach between (1) and (2), as in [20]. Gate potentials are applied by enforcing Dirichlet boundary conditions onto the model. For the other boundaries, a homogeneous Neumann boundary condition (∇φ·n=0, where n is the outward-pointing unit normal vector), is used. The obtained solution directly gives the charge distribution in graphene under the gate potential (without contacts present directly on the graphene), since the scenario describes the electrostatic case without any current flowing. 

In this work, Equation (2) has been discretized using the Finite Element Method (FEM) with a triangular mesh and with scalar (nodal) shape functions and is solved iteratively together with Equation (1), by using successive under-relaxation method as in [20,23]. Equation (2) is strongly coupled with Equation (1), where the potential is one coupling channel, and the concentration of carriers is the other one. In each iteration, for a given potential φ, carrier densities are computed by (1), and then they are transferred into the Poisson’s Equation (2) to update the potential distribution again. This iterative scheme continues until the potential distribution stabilizes. The model in Figure 1a also assumes that graphene layer is in contact with a source of carrier, which does not affect the Fermi level position and does not cause doping of the graphene. 

In addition to the analysis of gating on graphene, the contact doping and Fermi level pinning due to the contact of metals on graphene [24] can also be incorporated into this model, by applying additional Dirichlet boundary conditions as in Figure 1c. Since the potential φ is directly related to the Fermi level energy of graphene ($E_{F}$), it is possible to enforce the Fermi level pinning directly, by applying additional Dirichlet boundary conditions for the potential under equilibrium, and this leads to carrier doping due to the metal contacts. With these results, it is possible to visualize built-in fields, calculate the conductance of a device, and, ultimately, design contacts and dielectrics to find the ideal operation ranges for the gate voltages.

### 2.2. Hydrodynamic Charge Transport in Graphene

#### 2.2.1. Hydrodynamic Transport Model

The accurate analysis of semiconductor devices requires advanced models for the charge carrier transport. The traditional drift-diffusion models (DDM) start losing their validity as the operation frequencies increase [10], and the oscillation periods of the carriers become comparable with the momentum and energy-relaxation-time constants. Therefore, hydrodynamic models (HDM) have been employed to solve mass, momentum, and energy conservation equations for the carriers in the semiconductors, to provide better accuracy. Especially for reaching THz operation speeds and developing sub-micron devices, modeling inertia effects and ballistic transport with HDM is vital [17,18,25,26,27,28,29,30]. Applying a number of moment-expansion operations on the Boltzmann Transport Equation (BTE) would lead to transport models with increasing accuracy and complexity. One of the widely accepted set of equations for HDM are given for unipolar charge transport (assuming only electrons are present), as in (4)–(6).

(4) $$\frac{\partial n_{e}}{\partial t}+\nabla \cdot \left(n_{e}v\right)={\left(\frac{\partial n_{e}}{\partial t}\right)}_{c}\;,$$

(5) $$\frac{\partial p}{\partial t}+v\nabla \cdot p+p\cdot \nabla v=-qn_{e}E-\nabla \left(n_{e}k_{B}T_{n}\right)+{\left(\frac{\partial p}{\partial t}\right)}_{c}\;\;,$$

(6) $$\frac{\partial w}{\partial t}+\nabla \cdot \left(vw\right)=-qn_{e}v\cdot E-\nabla \cdot \left(vn_{e}k_{B}T_{n}\right)+\nabla \cdot \left(\kappa \nabla T_{n}\right)+{\left(\frac{\partial w}{\partial t}\right)}_{c}\;,$$

where the unknowns of the system are electron concentration $n_{e}$, momentum density of electrons p, and the energy density of electrons w. Additionally, κ stands for the heat-conduction coefficient ($\kappa =\kappa_{0}\mu_{n0}nk_{B}^{2}T_{0}$ as in the Wiedemann–Franz law), $T_{n}$ is the carrier temperature, v stands for the electron velocity, and E is the electric field (E=−∇φ) that exerts electric force on the electrons. A set of additional equations are given to relate the parameters as $p=m_{e}nv$, and $w=\frac{3}{2}nk_{B}T+\frac{1}{2}m_{e}n{\left|v\right|}^{2}$, where $m_{e}$ is the effective carrier mass. The complementary collision terms can be written as ${\left(\frac{\partial n_{e}}{\partial t}\right)}_{c}=0$, ${\left(\frac{\partial p}{\partial t}\right)}_{c}=-\frac{p}{\tau_{p}}$, and ${\left(\frac{\partial w}{\partial t}\right)}_{c}=-\frac{w-\frac{3}{2}nk_{B}T_{0}}{\tau_{w}}$, where $\tau_{p}$ and $\tau_{w}$ represent the momentum and energy relaxation times, respectively, and $T_{0}$ stands for ambient temperature. 

By assuming the geometry is 2-D, and separating the components of the vectorial quantities, this set of HDM equations can be written in a more compact way:

(7) $$\frac{\partial u}{\partial t}+\nabla \cdot \;F\left(u\right)=R\left(u\right),$$

(8) $$F\left(u\right)=a_{x}\left(v_{x}u+{\left[0,\;n_{e}k_{B}T_{n},\;0,\;v_{x}n_{e}k_{B}T_{n}\right]}^{T}\right)+a_{y}\left(v_{y}u+{\left[0,\;0,\;n_{e}k_{B}T_{n},\;v_{y}n_{e}k_{B}T_{n}\right]}^{T}\right),$$

(9) $$R\left(u\right)={\left[0,-en_{e}E_{x}-\frac{p_{x}}{\tau_{p}},-en_{e}E_{y}-\frac{p_{y}}{\tau_{p}},-en_{e}v\cdot E-\frac{w-\frac{3}{2}n_{e}k_{B}T_{0}}{\tau_{w}}+\nabla \cdot \left(\kappa \nabla T_{n}\right)\right]}^{T},$$

for the unknown vector $u={\left[n_{e},\;p_{x},\;p_{y},\;w\right]}^{T}$. Solutions of this set for conventional semiconductors have already been demonstrated by the use of DGTD-FEM in 1-D [25] and 2-D [18]. However, due to the exotic properties of graphene, direct application of this model on graphene is challenging. 

#### 2.2.2. Graphene Properties and Parameters

The hydrodynamic properties of charge transport in graphene have been demonstrated multiple times, such as its viscosity effects [8,10], Poiseuille flow [9], etc. Moreover, macroscopic models based on the Boltzmann equation are developed for the analysis of dynamic effects in graphene, such as thermal transport [31]. However, a generalized HDM solver to model charge-carrier transport has been missing. The HDM-transport solver would also complete the charge-transport analysis in graphene under non-equilibrium conditions, when it is coupled with the nonlinear Poisson’s solver described in the previous section. In other words, the full HDM solver, as described in (7)–(9), can be coupled with (2) and (3) to provide full analysis. Another approach could also be solving (2) and (3) initially to obtain the Fermi level in the graphene sheet, due to contact doping and gating, and (7)–(9) can be solved together with Poisson’s equation for the transport analysis, assuming contact doping stays steady. However, the introduced set of HDM Equations (7)–(9) are derived for traditional semiconductors with well-defined effective carrier mass. Additionally, for traditional semiconductors, the momentum and energy-relaxation mechanisms are well understood, and the corresponding time constants can be found in the literature. Therefore, in order for HDM transport to be applied on graphene, material specific parameters, carrier effective mass, saturation velocity, momentum, and energy-relaxation-time constants need to be known. As is widely known, one of graphene’s extreme properties is its exceptional carrier mass, due to its conical Dirac band structure. This massless characteristic of electrons and holes also results in very high mobility and electrical conductivity, making graphene a unique material. In this work, we follow the classical approach given in [32], without invoking the Dirac equation to obtain an average effective electron (and also hole) mass in graphene:

(10) $$\bar{m^{*}}=\frac{2E_{F}}{v_{F}^{2}},$$

where $v_{F}$ denotes Fermi velocity ($=8.96\times 10^{4}\;m/s$). For $E_{F}=0$, average effective carrier mass is also given with the following formula:

(11) $$\bar{m^{*}}=\frac{\left(4\ln2\right)k_{B}T}{v_{F}^{2}},$$

Another very important parameter for hydrodynamic transport is the momentum-relaxation-time constant that reveals itself in the right-hand-side collision term of the momentum-conservation Equation (5). This parameter is primarily dependent on the scattering mechanisms taking place, and for high-temperature applications, phonon scattering would be the primary scattering mechanism. The momentum-relaxation-time constant for phonon scattering in graphene as a function of Fermi level, phonon velocities, mass density, and acoustic deformation potential can be obtained by following the formalism in [33,34]. For T=300 K and $E_{F}=100\;\mathrm{meV}$, $\tau_{p}$ corresponds to 0.27 ps, while it can potentially reach up to 2 ps for the undoped graphene with a Fermi level near the Dirac point.

Moreover, carrier-saturation velocity also plays a significant role for the transport and can be written as [35,36]:

(12) $$v_{sat}=v_{F}\frac{\hbar \omega_{OP}}{E_{F}},$$

where $\hbar \omega_{OP}≅200\;\mathrm{meV}$ is the inelastic optical phonon (OP) scattering rate, observed when SiO_2_ is used as the gate-insulator material.

The last missing parameter for the HDM of graphene is energy-relaxation time that appears in the collision term of the energy conservation Equation (6). For this work, it is taken as 1 ps for high-temperature operation [37,38]. Having obtained all the material-related transport parameters for graphene, the HDM set can be solved, and the hydrodynamic transport effects can be observed with the appropriate set of boundary conditions.

## 3. Results

**Figure 2:**
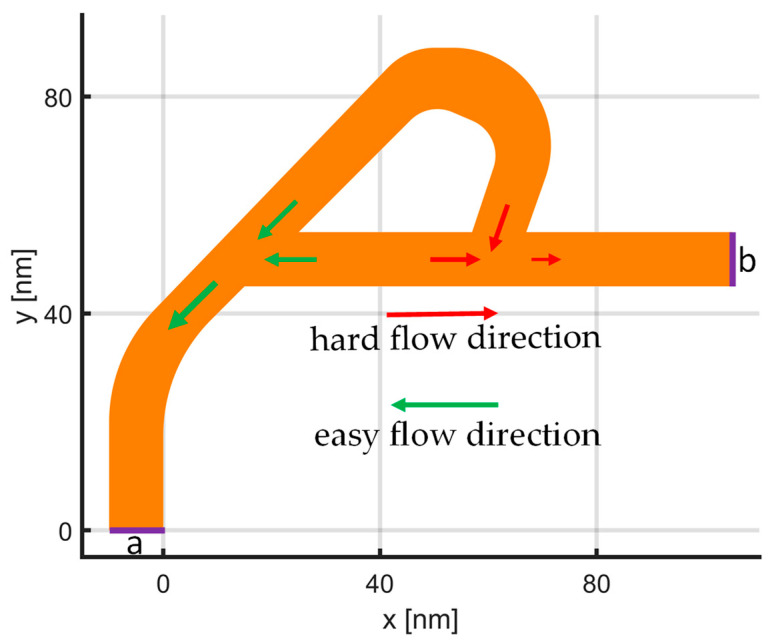
Geometry of 2-D graphene Tesla valve. The orange area is the full 2-D computation domain. Ohmic contacts are depicted with “a” and “b”, the charge carriers flow depending on the applied potential, either from “a” to “b” or from “b” to “a”. The boundaries are insulating, except for the ohmic-metallic contacts. Hard and easy flow directions are given by the arrows.

In order to test and analyze the developed 2-D hydrodynamic solver for graphene, a 2-D device geometry, as in Figure 2, is chosen. The orange part in Figure 2 denotes the graphene-based device as well as the full computation domain for the HDM simulation. The geometry is inspired from a mechanical device designed by Nikola Tesla, also referred to as a Tesla valve [39]. The main purpose and advantage of this device is that when a fluid flows through this device, it encounters different resistances depending on the flow direction. In other words, in one direction, (from a to b in Figure 2) the fluid is divided into two branches, and they recombine again with a large angle (opposing the main flow direction) creating whirlpools, loss of energy, and higher resistance for the flow. However, for the other direction (fluid flow from b to a), the flow again is divided into two branches, but it recombines later with a smoother angle without causing too much loss of energy. The authors of [10] have fabricated a similar Tesla-valve-like graphene device operating at low temperature, based on this phenomenon, where electron flow is more resistive in one direction with diode-like electrical rectification. Since a commercial hydrodynamic carrier transport solver for graphene is not available, the authors of [10] have performed simulations using fluid-dynamics solvers to show the effects qualitatively and also to verify their experimental findings. In this work, we also choose a similar structure with smaller dimensions to model the hydrodynamic charge transport in graphene at room temperature. 

### 3.1. Rectification: Tesla Valve 

The developed HDM solver for graphene is applied for the device geometry given in Figure 2, which represents the fluidic device, called a Tesla valve, in nanoscale for electron transport in graphene. In order to observe the pure viscosity effects and the working principles of a Tesla valve, a homogeneous Fermi level distribution is assumed on the graphene, which can be provided by gating from underneath. Moreover, ideal ohmic contacts without contact doping effects are employed for the boundary conditions at the “a” and “b” boundaries in Figure 2. Assuming homogeneous $E_{F}=100\;\mathrm{meV}$ on the graphene layer, a unipolar HDM solver (assuming only electrons exist) is employed, together with the Poisson’s equation to simulate the structure. The electric field (driving force for the carriers) between the ideal ohmic contacts are obtained by solving the Poisson’s equation, with respective Dirichlet boundary conditions. In other words, the model (7)–(9) is coupled with a Poisson’s equation solver in lateral dimensions, for the computation of the hydrodynamic charge carrier transport in graphene. This analysis is done independently of the transverse potential computation, thanks to the homogeneous Fermi level assumption. For a more accurate analysis, Poisson’s equation can be considered in 3-D, to account for both lateral and transverse variations at the same time.

The narrow width, in the order of 10 nm, of the graphene device in Figure 2, might also lead to edge effects and confinement effects, which could potentially change the electron velocities [31,40,41]. In this work, we focus on macroscopic carrier transport and collective carrier motion, and the mentioned effects are omitted by using average values. However, thanks to the flexible nature of HDM equations, the varying carrier velocity and different phonon-scattering times can also be incorporated into the model for more accurate graphene-nanoribbon simulations.

There are two types of boundary conditions used in this structure, the first one is the ohmic contact condition on the boundaries ‘a’ and ‘b’, to make sure the potential is applied on electrons, so they can enter and leave the structure without resistance. The other boundary condition is applied on the edges of the Tesla valve structure. For that purpose, an isolation boundary is used to eliminate the possible flow outside of the computational domain. This isolation condition is implemented weakly, by assuming zero velocity just outside of the graphene device. In other words, usage of the DGTD method allowed us to assign zero velocity for the carriers on the “*ghost elements*”, just outside the device domain, and their presence is felt through the numerical-flux term in the DGTD-FEM formalism [18]. For the numerical flux, local Lax Friedrichs flux has been utilized [42], and time steps as small as 0.01 fs are needed to provide the stability, due to high carrier velocities and concentrations. The developed solver takes around 290 ms CPU time for the computation of each time step on an Intel i7-7700, requiring less than 100 MB memory for the device geometry in Figure 2, with triangular mesh consisting of 21,000 triangular elements. To sum up, for simulating 1 ps of dynamic transport, usually there is a required duration for the steady state to be formed: the solver computes 100,000 time steps. Thanks to the discontinuous nature of the employed DGTD method, a parallel approach can be developed for the next-generation implementation, and the resulting computation cost and required CPU time can be relieved. This would also lead to acceptable computation durations for the simulations of bigger structures and devices made out of graphene.

**Figure 3:**
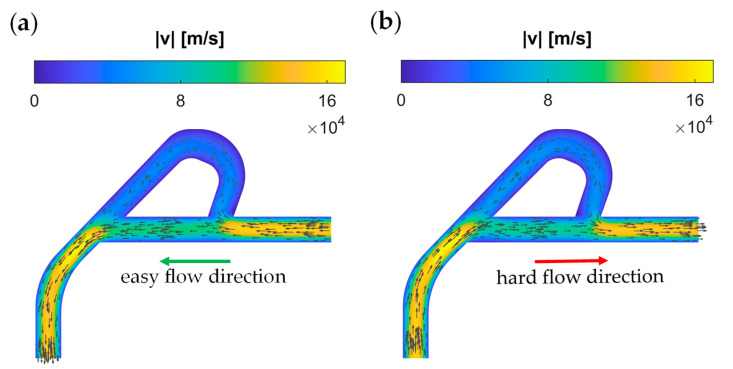
Hydrodynamic electron flow demonstrated in graphene Tesla valve. (**a**) Electron flow from right contact to the left, due to higher (50 mV) potential on the left; easy and less-resistive direction for the hydrodynamic flow. (**b**) Electron flow toward the right contact, harder and more resistive path for electrons, due to viscosity effects and momentum-loss, due to the large angle at the connection of the branches. The small difference in velocities causes the rectification effect (see the diodicity analysis below in the text).

**Figure 4:**
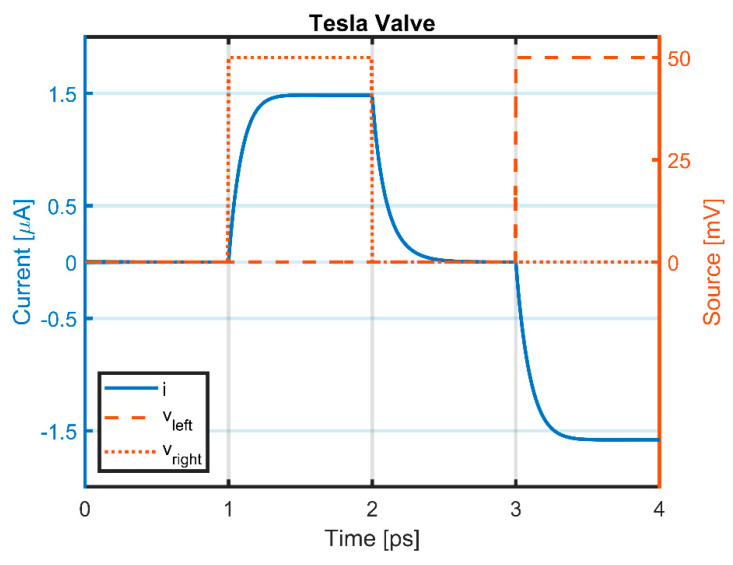
Transient voltage and current profiles of the Tesla valve during “easy” and “hard” flow modes. Between 1–2 ps, a bias in the ‘hard’ flow direction is applied, i.e., the right contact is biased to 50 mV while the left contact is kept grounded. Between 3–4 ps, the biased is reversed to facilitate current in ‘easy’ direction. The computed current level between 3–4 ps is higher than the current levels observed in the ‘hard’ flow direction between 1–2 ps.

Electron flow directions and velocities can be seen in Figure 3, for the cases where the contact on the right is grounded, and 50 mV is applied to the left contact, and vice versa. As it can be seen from Figure 3a, electrons meet again without too much resistance, when they flow in the easy direction, whereas electrons with opposing flow directions cause loss of energy in the hard-flow direction, as can be seen from Figure 3b. This different resistance experienced by the electrons, depending on the flow direction, also shows itself in the total current flowing through the device. Figure 4 depicts the transient current vs. time and the transient potential on the left and right contacts. When the left contact is kept at 50 mV, electrons flow toward the left, and they generate a higher absolute current, compared to the case where the right contact is kept at 50 mV and the left one is grounded. The diodicity parameter can be defined for this device similar to [10] as

(13) $$D=\frac{R_{hard}}{R_{easy}}=\left|\frac{i_{easy}}{i_{hard}}\right|,$$

For the same level of bias applied from the contacts in both the easy- and hard-flow cases, the diodicity yields to D=1.0655. Higher diodicities can be easily achieved by cascading several of these Tesla valves elements.

### 3.2. Poiseuille Flow

**Figure 5:**
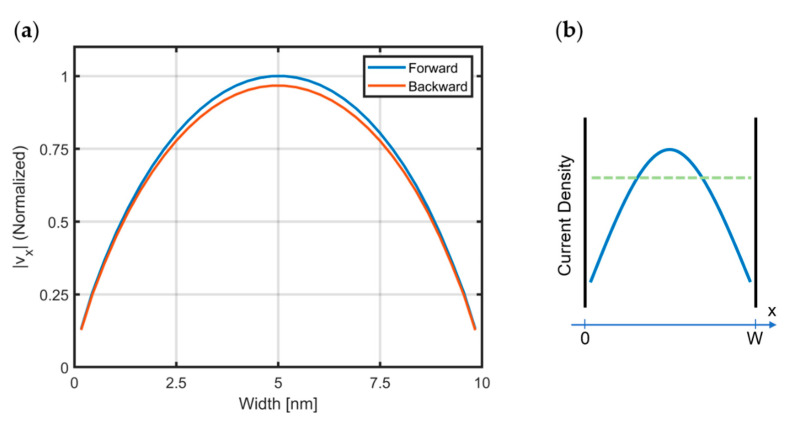
Velocity profile of electrons: (**a**) computed on the straight part of the Tesla valve geometry in Figure 2 near the right contact “b”. The electrons demonstrate Poiseuille flow as their velocity is maximum in the middle of the narrow channel and approach to zero near isolation-boundary condition of HDM. Moreover, the ‘easy’ flow direction predicts higher current values, as expected. (**b**) A schematic showing local-current-density profiles in a narrow channel (of width W) for conventional ohmic transport (green) and viscous Poiseuille flow (blue).

The classical Poiseuille flow is a well-known phenomenon, due to hydrodynamic effects and the collective motion that occurs when fluids flow in a long duct, such as a pipe. It has also been shown that under certain circumstances, the electrons in graphene also demonstrate the Poiseuille flow, while carrying a current [8,9]. In order to account for this effect, modified isolation boundary conditions have been used in the HDM solver, by enforcing the velocity of carriers to be zero, just outside the computational domain (in the ‘*ghost*’ elements). Therefore, the velocity profiles in the narrow channels, as in Figure 5, can be obtained; this ensures that the current is the maximum in the middle of the narrow graphene channels, and the electron flow tries to avoid the edges. Moreover, the current levels are higher when the electrons are flowing in the easier direction, which is labeled as ‘forward’, and maximum of the current is less in the ‘backward’ or harder-flow direction.

## 4. Discussion

Two separate solvers to model and simulate the charge behavior in graphene have been developed. The first simulator combines the nonlinear Poisson’s equation with the Thomas–Fermi equation in graphene, for the computation of the Fermi level and carrier distribution under the gate potential and contact doping, due to the metal layers. An example scenario is given in Figure 1 that analyzes the static fields in the transverse direction. The second solver considers the dynamic behavior of electrons and holes in graphene, by solving mass, momentum, and energy-conservation equations together. The proposed transport solver reveals the hydrodynamic charge transport in the lateral dimension and is qualitatively validated by simulating a graphene Tesla valve device. The modified HDM in graphene can account for the viscosity effects and collective carrier motion, and this enables modeling the hydrodynamic effects occurring in graphene-based devices, together with the Poisson’s solver. Thanks to the modified isolation boundary condition, the Poiseuille flow can also be modeled, which is effective especially for the narrow carrier flow channels. Finally, the results obtained by the proposed models match, qualitatively, with the experimental findings of the literature for both the electrostatic case with contact doping and the hydrodynamic transport seen in the Tesla valve.

The developed solvers can be adopted for the development of next-generation graphene devices, especially for THz applications, by modeling and making use of the carrier inertia effects, viscosity effects, and possible ballistic charge transport. Furthermore, graphene nanoribbons (GNR) can also be modeled with this approach, by using appropriate carrier velocity and phonon-scattering times, emerging due to edge effects. Moreover, a parallel solver for the HDM can be implemented in the next generation, thanks to the employed DGTD algorithm. 

## Data Availability

The data presented in this study are available on request from the corresponding author.

## Supplementary Materials

The following supporting information can be downloaded at: https://www.mdpi.com/article/10.3390/ma15124141/s1, Figure S1: 2-D device geometry (cross-section) with two gates at the top and the bottom. Graphene is depicted as orange layer in the middle, the green layers represent dielectrics, the gate contacts are shown in golden, and Dirichlet conditions are applied to those boundaries for solving the nonlinear Poisson’s equation; Figure S2. Fermi level and charge carrier profile on graphene layer with respect to changing top gate potentials while the bottom gate is kept grounded. (a,c,e,g,i) show the Fermi level profile for the varying gate potentials (50 mV, 100 mV, 500 mV, 1 V, 5 V respectively) computed by using Equations (S1) and (S4). The full Thomas-Fermi dynamics in (S1) considers temperature dependence (black curves) while (S4) assumes 0 K (purple curves). (b,d,f,h,j) depict the carrier distribution on the graphene layer for the varying gate potentials. Blue, orange and dashed black curves are obtained from the full model (1)–(3), the purple curve for the electron concentration assumes 0 K based on (S4). As the gate potential, and the Fermi level, increases the computed distributions from both approaches come closer to each other, moreover hole concentration becomes negligible implying the 0 K approach could be used and poly-logarithm function can be avoided.
