# Supplementary material for: Modeling Hydrodynamic Charge Transport in Graphene
Source: Materials (Basel). 2022 Jun 10;15(12):4141. doi: 10.3390/ma15124141 (PMC9228317; doi:10.3390/ma15124141)
Supplement: Supplementary file 1 [file materials-15-04141-s001.zip › materials-1748394-supplementary.pdf]

Supplementary Material

Nonlinear Electrostatic Gating

In this supplementary material two modeling approaches for the gating of graphene have been compared with each other. The first model is based on the formulation described in the article with the Equations (S1)–(S3). This model considers the temperature dependence as well and uses Equation (S1) with the poly-logarithm function for the relation between Fermi level and the carrier concentration [1,2]. These equations are solved iteratively [3] until a convergence is reached to model gating of graphene. The results of the model have been demonstrated in the article.

$$n_e^{2D}(E_F) = -\frac{2}{\pi}\left(\frac{k_B T}{\hbar v_F}\right)^2 Li_2\left(-e^{\frac{E_F}{k_B T}}\right), \tag{S1}$$

$$\nabla(\varepsilon\nabla\varphi) = -\rho(\varphi), \tag{S2}$$

$$\rho(\varphi) = \begin{cases} q[n_h^{2D}(\varphi) - n_e^{2D}(\varphi)]/t_{gr} & \text{, within graphene} \\ 0 & \text{, otherwise} \end{cases} \tag{S3}$$

For some applications, especially when the Fermi levels are far from Dirac point, it is possible to avoid the poly-log function by only using the Fermi level carrier concentration relation for the 0 K case as in Equation (S4). For high levels of Fermi level, the hole concentration also becomes negligible, and therefore total charge density can be written by only using the electron concentration. Similarly, for negative values of Fermi level (below the Dirac point), electron concentration can be neglected and hole concentration can be obtained using (S4) [4,5].

$$n_e^{2D}(E_F) = \frac{E_F^2}{\pi(\hbar v_F)^2} \tag{S4}$$

These two models are compared for the structure given in Figure S1 by varying the gate potential on the top gate. The comparative study, as shown in Figure S2, suggests that as the gate potential and the Fermi level increase, both approaches yield similar results, and the hole concentration becomes negligible and the effect of temperature consideration on carrier concentration is very little. However, for the small gate potentials, the relation in (S1) needs to be considered, replacing (S1) with (S4) would yield reduced carrier concentrations levels and higher Fermi levels.

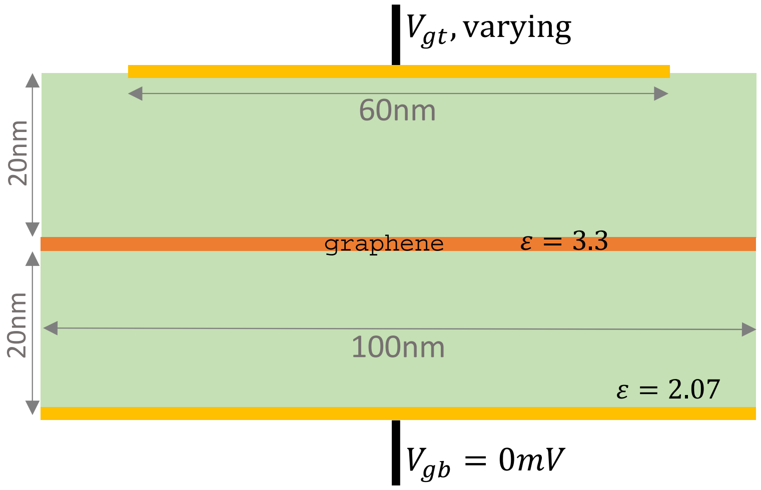

Figure S1. 2-D device geometry (cross-section) with two gates at the top and the bottom. Graphene is depicted as orange layer in the middle, the green layers represent dielectrics, the gate contacts are shown in golden, and Dirichlet conditions are applied to those boundaries for solving the nonlinear Poisson's equation.

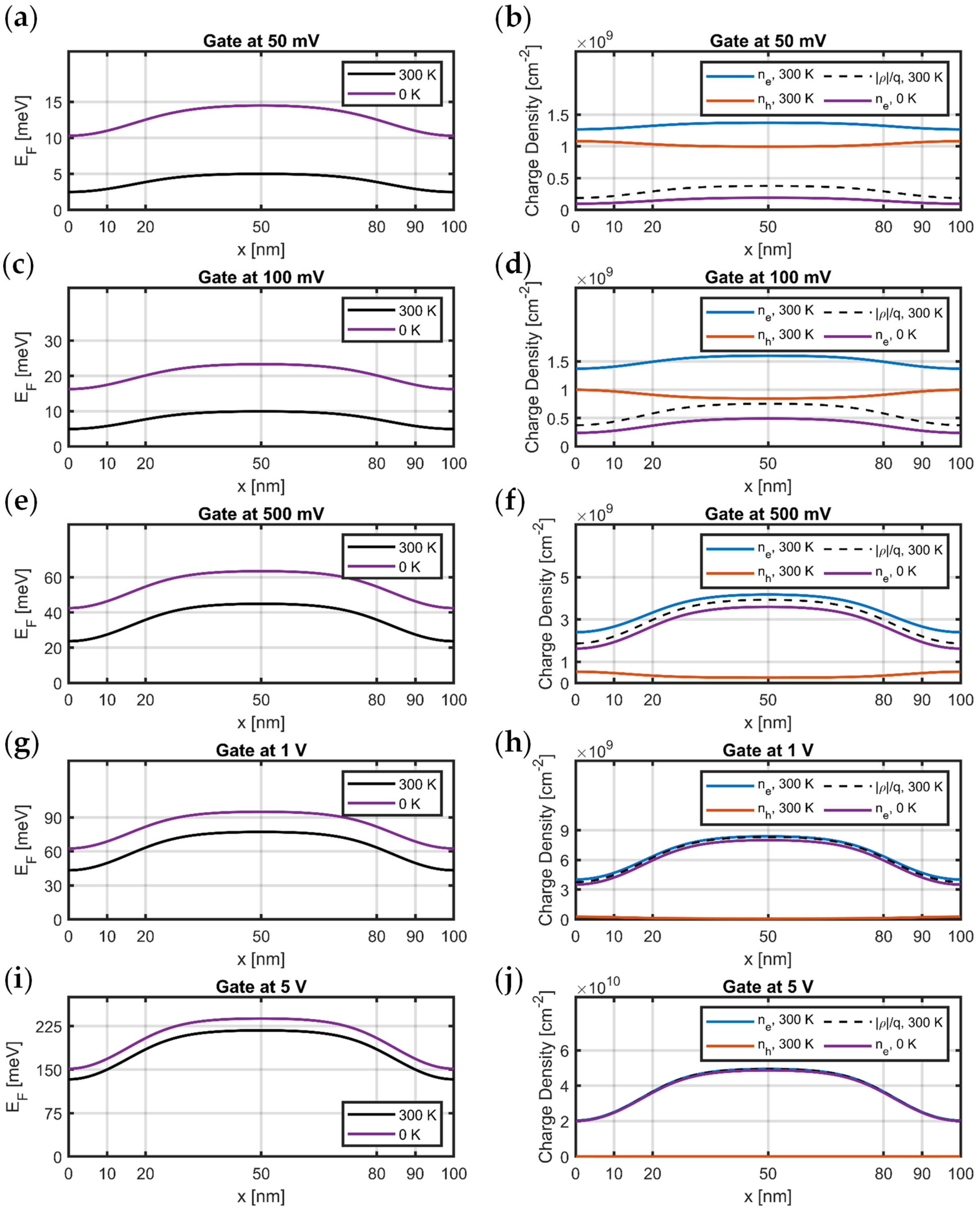

Figure S2. Fermi level and charge carrier profile on graphene layer with respect to changing top gate potentials while the bottom gate is kept grounded. (a,c,e,g,i) show the Fermi level profile for the varying gate potentials (50 mV, 100 mV, 500 mV, 1 V, 5 V respectively) computed by using Equations (S1) and (S4). The full Thomas-Fermi dynamics in (S1) considers temperature dependence (black curves) while (4) assumes 0 K (purple curves). (b,d,f,h,j) depict the carrier distribution on the graphene layer for the varying gate potentials. Blue, orange and dashed black curves are obtained from the full model (1)–(3), the purple curve for the electron concentration assumes 0 K based on (S4). As the gate potential, and the Fermi level, increases the computed distributions from both approaches come closer to each other, moreover hole concentration becomes negligible implying the 0 K approach could be used and poly-logarithm function can be avoided.

References

1. Nastasi, G.; Romano, V. A full coupled drift-diffusion-Poisson simulation of a GFET. *Communications in Nonlinear Science and Numerical Simulation* **2020**, *87*, doi:10.1016/j.cnsns.2020.105300.
2. Zebrev, G. Graphene field effect transistors: Diffusion-drift theory. *Physics and Applications of Graphene-Theory* **2011**, 475-498.
3. Andrijauskas, T.; Shylau, A.A.; Zozoulenko, I. Thomas–Fermi and Poisson modeling of gate electrostatics in graphene nanoribbon. *Lithuanian Journal of Physics* **2012**, *52*.
4. Liu, M.-H. Gate-induced carrier density modulation in bulk graphene: theories and electrostatic simulation using Matlab pdetool. *Journal of Computational Electronics* **2013**, *12*, 188-202.
5. Yu, Y.-J.; Zhao, Y.; Ryu, S.; Brus, L.E.; Kim, K.S.; Kim, P. Tuning the graphene work function by electric field effect. *Nano letters* **2009**, *9*, 3430-3434.
